# Genome-wide analyses of SWEET family proteins reveal involvement in fruit development and abiotic/biotic stress responses in banana

**DOI:** 10.1038/s41598-017-03872-w

**Published:** 2017-06-14

**Authors:** Hongxia Miao, Peiguang Sun, Qing Liu, Yulu Miao, Juhua Liu, Kaixing Zhang, Wei Hu, Jianbin Zhang, Jingyi Wang, Zhuo Wang, Caihong Jia, Biyu Xu, Zhiqiang Jin

**Affiliations:** 1 Key Laboratory of Tropical Crop Biotechnology, Ministry of Agriculture, Institute of Tropical Bioscience and Biotechnology, Chinese Academy of Tropical Agricultural Sciences, Haikou, 571101 China; 2 Key Laboratory of Genetic Improvement of Bananas, Hainan Province, Haikou Experimental Station, Chinese Academy of Tropical Agricultural Sciences, Haikou, 570102 China; 3 CSIRO Agriculture and Food, GPO Box 1600, ACT 2601 Canberra, Australia; 4 Department of Agriculture, Hainan University, Haikou, 570228 China

## Abstract

Sugars Will Eventually be Exported Transporters (SWEET) are a novel type of sugar transporter that plays crucial roles in multiple biological processes. From banana, for the first time, 25 *SWEET* genes which could be classified into four subfamilies were identified. Majority of *MaSWEETs* in each subfamily shared similar gene structures and conserved motifs. Comprehensive transcriptomic analysis of two banana genotypes revealed differential expression patterns of *MaSWEETs* in different tissues, at various stages of fruit development and ripening, and in response to abiotic and biotic stresses. More than 80% *MaSWEETs* were highly expressed in BaXi Jiao (BX, *Musa acuminata* AAA group, cv. Cavendish), in sharp contrast to Fen Jiao (FJ, *M*. *acuminata* AAB group) when pseudostem was first emerged. However, *MaSWEETs* in FJ showed elevated expression under cold, drought, salt, and fungal disease stresses, but not in BX. Interaction networks and co-expression assays further revealed that *MaSWEET*-mediated networks participate in fruit development signaling and abiotic/biotic stresses, which was strongly activated during early stage of fruit development in BX. This study provides new insights into the complex transcriptional regulation of *SWEETs*, as well as numerous candidate genes that promote early sugar transport to improve fruit quality and enhance stress resistance in banana.

## Introduction

In plants, sugars not only provide energy, essential signal molecule components (carbon), and metabolic intermediates for plant growth and development, but also play crucial roles in both abiotic and biotic stress responses^1–5^. Sugar transport from source- to sink cells is controlled by multiple transporters, i.e. sucrose transporters (SUT) and monosaccharide transporters^3, 6–8^. Several studies revealed a constantly low level of SUT expression and saturable sucrose transport kinetics, indicating that SUTs may not be the key rate-limiting factor for sucrose transport but additional transporters are responsible for sucrose allocation across the membrane^9–14^. Recently, a new class of sugar transporter, the Sugar Will Eventually be Exported Transporters (SWEET), was identified in *Arabidopsis thaliana* ^15^. Unlike SUTs which contain 12 transmembrane domains (TMD) connected by hydrophilic loops and catalyze sucrose/H^+^-coupled transport^3, 13, 14, 16^, SWEET proteins contain 7 TMD connected by a PQ-loop-repeat^15, 17, 18^ and function as key transporters for sucrose, hexose, and fructose along a concentration gradient^19^.

To date, genome-wide analysis has identified variable number of *SWEET* genes in plants, including 17 in *A*. *thaliana* ^15^, 21 in rice (*Oryza sativa*)^20^, 23 in sorghum (*Sorghum bicolor*)^21^, 52 in soybean (*Glycine max*)^22^, 35 in potato (*Solanum tuberosum*)^23^, 29 in tomato (*Solanum lycopersicum*)^18^, 33 in apple (*Malus domestica*)^24^, and 17 in grape (*Vitis vinifera*)^4^. Phylogenetic analysis revealed that SWEETs could be classified into four subfamilies (Clades I-IV) in *A*. *thaliana*, *O*. *sativa*, *V*. *vinifera*, *S*. *lycopersicum*, and *M*. *domestica* ^14, 18, 22, 24^. SWEETs in Clades I and II preferentially transport hexoses^15, 25^, while those in Clade III are sucrose transporters^25, 26^. Clade IV contains relatively less number of *SWEET* genes compared to other Clades and has been shown to largely consist of vacuolar transporters controlling the flux of fructose across the tonoplast^27, 28^. Biochemical and functional analyses have shown SWEETs’ involvement in multiple essential plant biological processes, including growth, senescence^26, 28^, seed and pollen development^15, 29, 30^, environmental adaptation^20^, and host-pathogen interactions^4, 15^. For example, in *A*. *thaliana*, *AtSWEET17* knockout mutants exhibited a reduced root growth phenotype^28^, and *AtSWEET15* was upregulated ~22-fold in senescent leaves possibly involved in the regulation of carbohydrate mobilization^31^. *AtSWEET8* contributes to pollen viability, suppression of which was shown to reduce starch content in pollen grains and cause male sterility^15^. *AtSWEET16*-overexpressing plants were shown to display significant alterations in sugar levels and developmental processes, like germination and growth^32^. Overexpression of *AtSWEET4* increased plant size^33^. In addition, expression of some *SWEET* genes can be induced by various stress conditions, including cold, salt, drought, and plant-pathogens^4, 15, 33, 34^. Previously, *AtSWEET4* expression was reported to enhance tolerance to freezing and drought stresses^4, 33^. *SISWEET15* in *S*. *lycopersicum* was identified as an important regulator of salt tolerance^34^. *Pseudomonas syringae* infection was shown to significantly induce the expression of *AtSWEET*-*4*, -*5*, -*7*, -*8*, -*10*, -*12*, and -15 in *A*. *thaliana* leaves^15^. In *V*. *vinifera*, *VvSWEET4* expression was reported to participate in the interaction with *Botrytis cinerea* ^4^. Taken together, these studies have revealed many important roles of the *SWEET* gene family in the regulation of plant growth, development, and responses to abiotic and biotic stresses.

Banana (*Musa acuminata* L.) is not only one of the most favored starchy fruits worldwide, but also an important staple food in some African and Latin American countries^35, 36^. Sugar transporters are extremely important for banana plant since sugars play an important role in starch accumulation and sugar content is also a major component of fruit quality and economic value. Being a large annual monocotyledonous herbaceous plant, banana is frequently affected or even destroyed by various abiotic and biotic stresses during growth and development. Investigations of key candidate genes involved in fruit development and response to different stress conditions based on complete genome sequences have been conducted in several fruit crops, but only few in banana^37–39^. Despite of its importance in fruit quality, plant growth and stress regulation, *SWEET* genes have not been studied in banana.

For the first time, we have conducted a genome-wide analysis of *SWEET* genes in banana, termed as *MaSWEET*, and analyzed their phylogenetic relationship, gene structure, protein motifs, spatial and temporal expression patterns, and expression in response to both abiotic and biotic stresses in two different *M*. *acuminata* varieties. In addition, we characterized interaction networks and co-expression patterns of *MaSWEETs* in early stages of fruit development and under abiotic and biotic stresses. This comprehensive study serve to facilitate our understanding of *MaSWEET* associated fruit development processes and stress responses, and provide a foundation for future studies of crop improvement involving *SWEETs*.

## Results

### Identification and phylogenetic analysis of banana *MaSWEET* family members

To identify all *MaSWEET* members in BaXi Jiao (BX) [*M*. *acuminata* L. AAA group, cv. Cavendish] and Fen Jiao (FJ) [*M*. *acuminata* AAB group] bananas, Hidden Markov Model searches using typical *SWEET* domains (PFAM: PF03083) as queries, as well as BLAST searches using *AtSWEET* and *O*. *sativa OsSWEET* sequences as queries, were performed in the banana genome database. After validating the *SWEET* domain using the CDD and PFAM databases, a total of 25 *MaSWEETs* were identified. *MaSWEETs* were named according to their respective orthologous genes in *A*. *thaliana*. The 25 predicted MaSWEET proteins varied from 171 (MaSWEET7b) to 333 (MaSWEET14j) amino acid residues in size, with relative molecular masses between 19.097 (MaSWEET7b) and 37.418 (MaSWEET14j) kDa, and isoelectric points ranging from 5.29 to 9.83 (Supplementary Table S1). The highly variable structure of MaSWEETs may suggest their potentially different functional roles in different biological processes or under different growth conditions.

**Figure 1:**
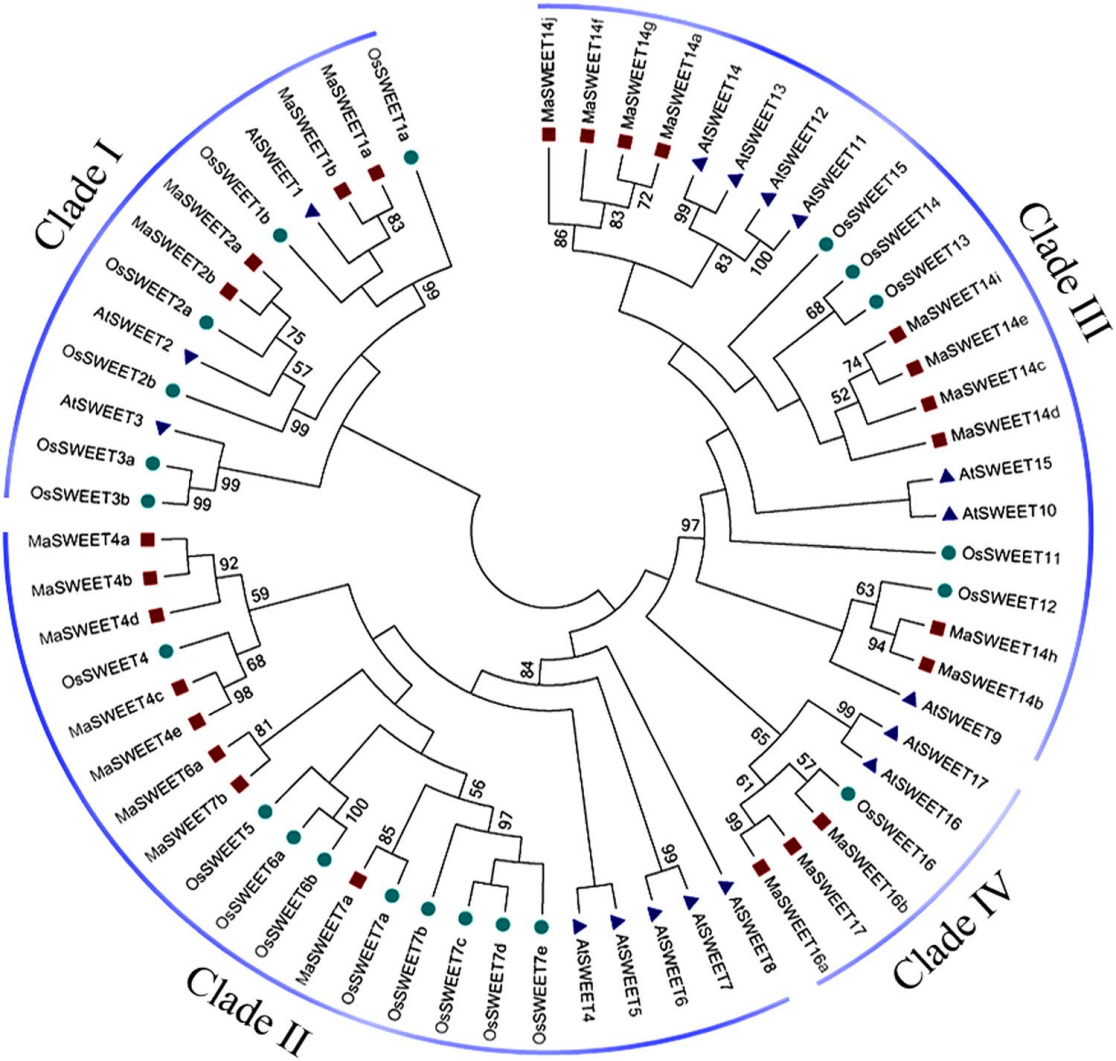
Phylogenetic analysis of the SWEETs from *Arabidopsis*, rice and banana. The Neighbor-joining tree was drawn using MEGA5.0 with 1000 bootstraps. Four subgroups were identified and classified as Clades I-IV.

To study the evolutionary relationships between SWEET family proteins, a neighbor-joining tree was generated by aligning 25, 17, and 21 SWEET proteins from banana, *A*. *thaliana*, and *O*. *sativa*, respectively, using ClustalX and MEGA5.0 software (Fig. 1). Based on the phylogenetic tree, all MaSWEET proteins were grouped into four Clades (I–IV); Clades II and III were large, with more than 8 MaSWEET members, whereas Clades I and IV contained less than 4 MaSWEET proteins, consistent to *A*. *thaliana* and *O*. *sativa* ^15, 20^. Distinct from other plant species, there were 10 *MaSWEET14* members in Clade III, while only 1 each in *A*. *thaliana* and *O*. *sativa* (Fig. 1). Besides, the Ka/Ks ratio of each duplicated gene pairs between AAA and AAB, AAA and Arabidopsis, and AAA and rice was calculated to estimate the molecular evolutionary rates (Supplementary Table S2). In AAA and AAB, the Ka/Ks ratios from 22 duplicated gene pairs were less than 1, while in other 3 duplicate gene pairs are more than 1. In AAA and rice, Ka/Ks ratios of 23 duplicated gene pairs were less than 1, only 1 duplicate gene pair is more than 1. Whereas in AAA and Arabidopsis, the Ka/Ks ratios from 11 duplicated gene pairs were less than 1, the other 14 duplicate gene pairs are more than 1.

### Gene structure and conserved motif analysis of banana *MaSWEETs*

**Figure 2:**
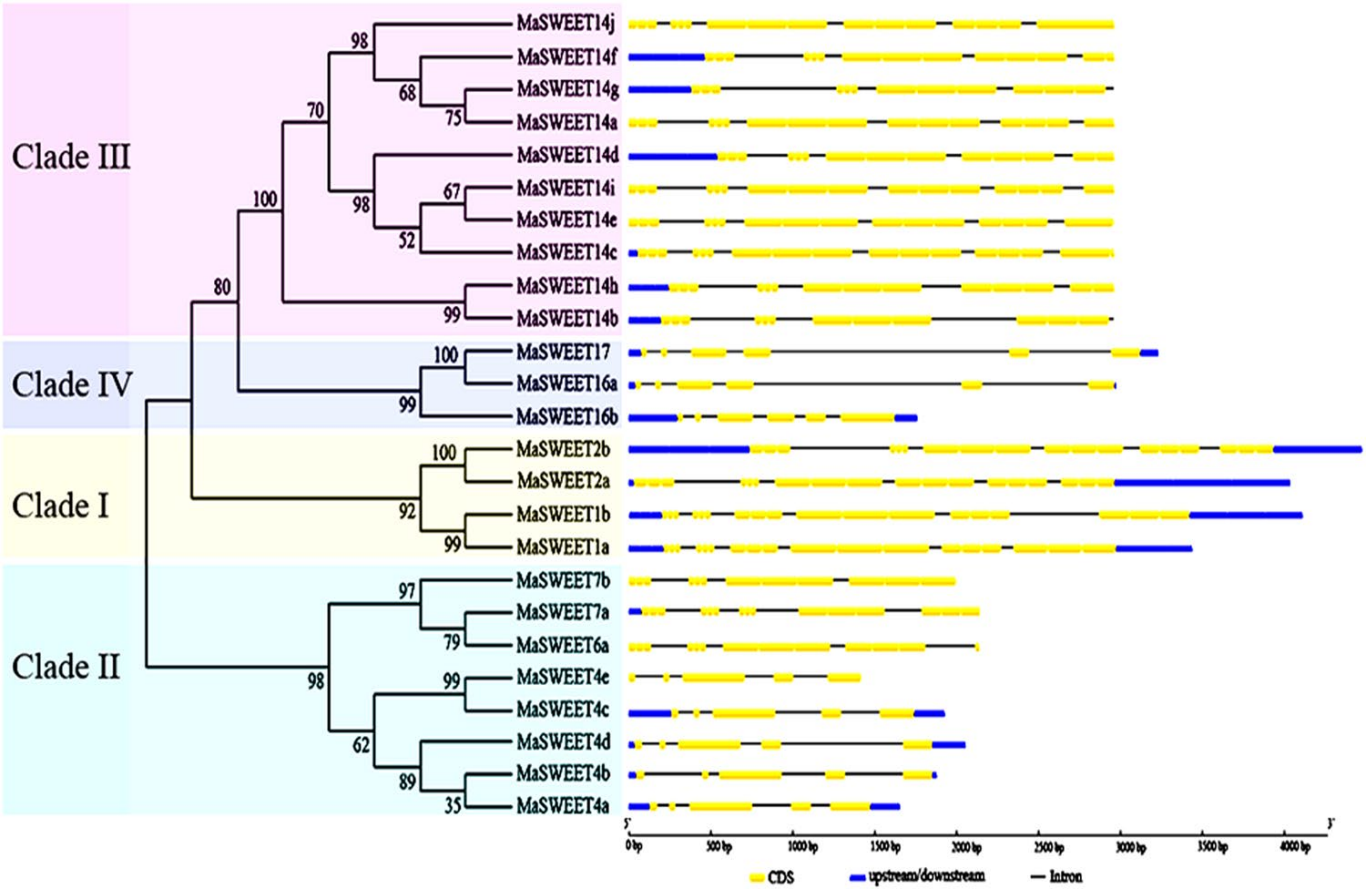
Gene structure analyses of *MaSWEETs*. Exon-intron structure analyses were performed using the Gene Structure Display Server database. Blue boxes indicate upstream/downstream; yellow boxes indicate exons; black lines indicate introns.

Evolutionary analysis further supported the classification of 25 *MaSWEETs* into four groups, in accordance with cluster analysis data (Fig. 2). The exon-intron structural evolution using Gene Structure Display Server software showed that the *MaSWEETs* contained 6 exons in Clades I and IV, 4–5 exons in Clade II, and 4–6 exons in Clade III. This suggests that *MaSWEETs* in the same group share similar exon-intron organizations.

**Figure 3:**
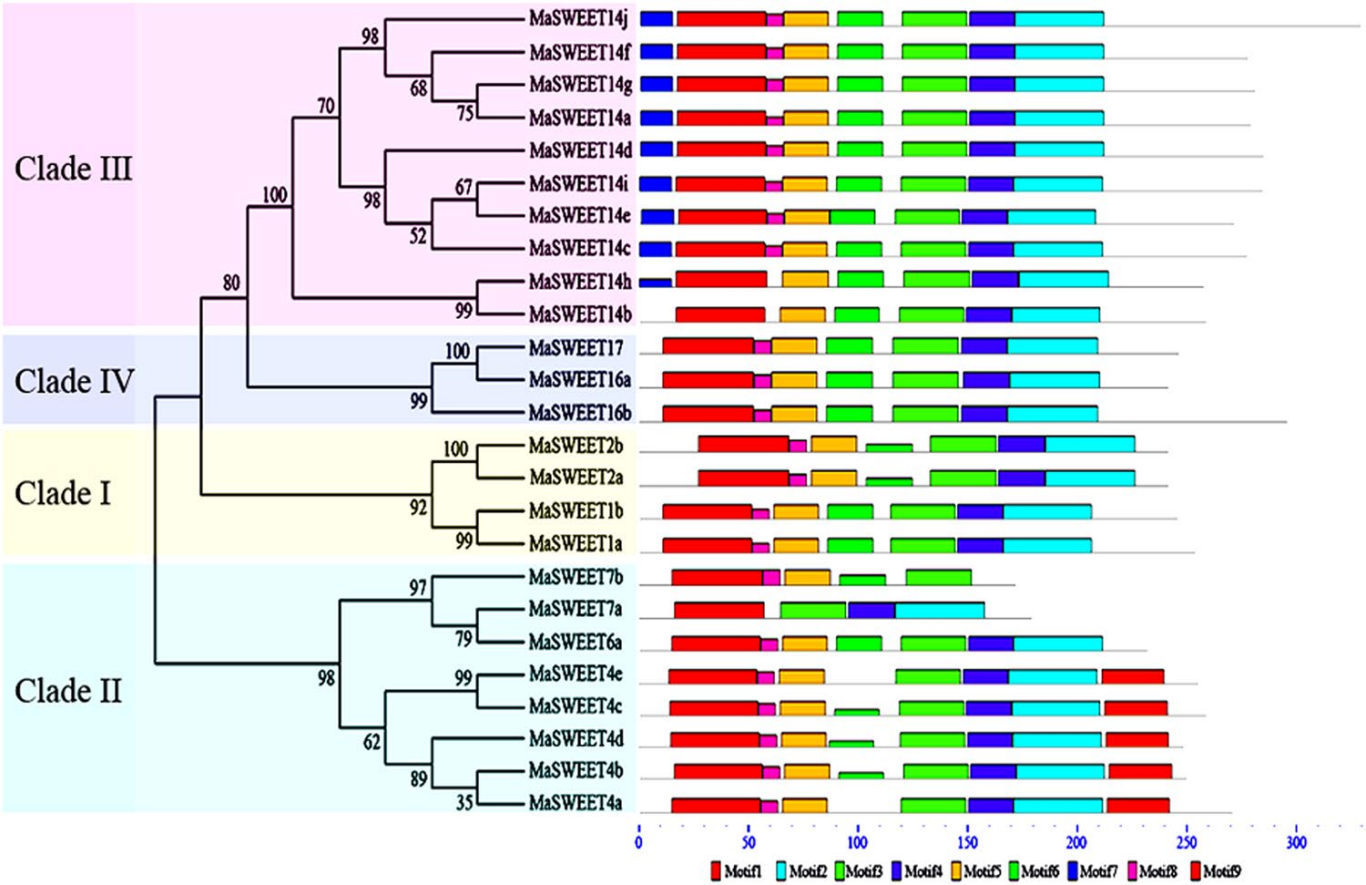
Phylogenetic and motif analyses of MaSWEET proteins. All proteins were identified by MEME database with the complete amino acid sequences of each MaSWEET identified. MaSWEETs were classified into Clades I-IV based on their phylogenetic relationships.

The conserved motif analysis and functional annotation prediction of banana *MaSWEETs* were then performed based on phylogenetic relationships (Fig. 3; Supplementary Table S3). A total of 9 conserved motifs in all 25 MaSWEETs were identified using MEME software and annotated with the InterPro database. Motifs 1–6 and 8 were annotated as 7 TMD helices, and all 25 identified MaSWEETs contained typical SWEET-type TMD helices. In particular, 7 TMD helices were found in all MaSWEETs in Clades I and IV and most in Clade II including MaSWEET-4a-e, and -6a and Clade III including MaSWEET-14a, -14c-g, -14i, and -14j. In Clade II, motif 5, 7, and 8 were lacking in MaSWEET7a, while motifs 2 and 4 were lacking in MaSWEET7b. In Clade III, motif 8 was found in neither MaSWEET-14b nor MaSWEET-14h. These results suggest that MaSWEETs clustered in the same group share conserved motifs which further supports the phylogenetic results.

### Expression patterns of *MaSWEETs* in different banana tissues

**Figure 4:**
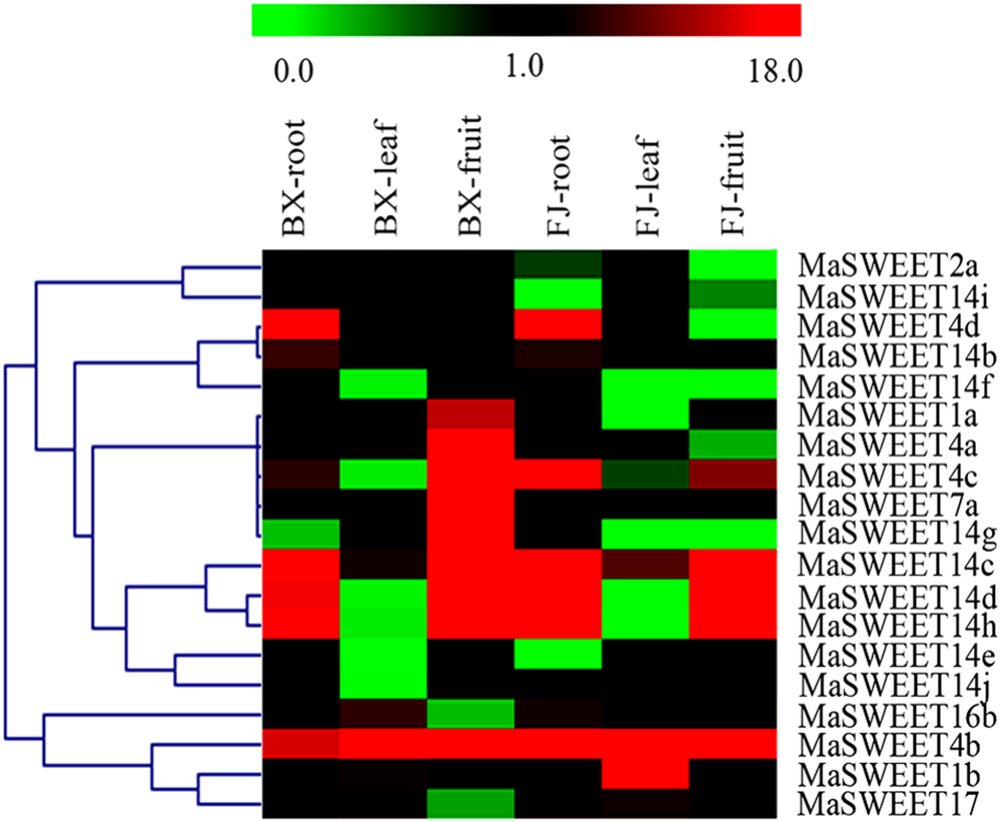
Expression of *MaSWEETs* in roots, leaves, and fruits of BX and FJ banana varieties. The heat map with clustering was created based on the FPKM value of the *MaSWEETs*. Differences in gene expression changes are shown in color as the scale.

To investigate the *MaSWEET* spatial expression patterns in banana, roots, leaves, and fruits of BX and FJ were sampled and subjected to transcriptional analysis. 19 (excluding *MaSWEET*-*2b*, -*4e*, -*6a*, -*7b*, -*14a*, and -*16a*) of the 25 *MaSWEETs* were expressed in at least one of the tested tissue in both varieties (Fig. 4 and Supplementary Table S4).

In BX genotype, 12 *MaSWEETs* (63%) were expressed in roots, 11 (58%) in leaves, and 11 (58%) in fruits. Of these *MaSWEET* genes, 5 (42%) were highly expressed [Reads Per Kb of exon model per Million mapped reads (FPKM) >10] in roots, 1 (9%) in leaves, and 9 (82%) in fruits. Additionally, *MaSWEET4b* exhibited high transcriptional expression (FRKM >14) in all tissues examined.

In FJ genotype, 14 (74%), 11 (58%), and 13 (68%) *MaSWEETs* were expressed in the roots, leaves, and fruit, respectively. Among them, 6 (43%), 2 (18%), and 5 (39%) *MaSWEETs* were highly expressed (FRKM > 10) in roots, leaves, and fruit, respectively. Furthermore, *MaSWEET4b* was highly expressed (FRKM > 63) in all organs tested.

Comparison of spatial expression profiles between the two banana varieties revealed 19 *MaSWEET* genes were expressed in BX compared to 17 in FJ. Moreover, similar spatial expression patterns of 5 *MaSWEET* genes (*MaSWEET*-*4b*, -*14b*, -*14c*, -*14d*, and -*14h*) were found in BX and FJ root and leaf. Significantly more *MaSWEET* genes (*MaSWEET*-*4a*, -*4c*, -*14c*, -*14d*, -*14g*, and -*14h*) were highly expressed in fruit than leaf in both genotypes. In contrast, some spatial expression patterns of *MaSWEET* were different between the two genotypes. In roots, for example, *MaSWEET4c* showed relatively lower expression (FPKM < 5.5) in BX compared to FJ (FPKM > 22.4). In fruit, 5 genes (*MaSWEET*-*1a*, -*4a*, -*4c*, -*7a*, and -*14g*) were highly expressed in BX (527 > FPKM > 13) in contrast to rather low expression in FJ (0 < FPKM < 5), suggesting *MaSWEET* members may play different roles in different organs in each banana variety. Furthermore, in all tested organs including roots, stems, and fruits, *MaSWEET4b* was highly expressed (FPKM > 10) in both BX and FJ, indicating a key role for this particular gene in all tissues, but further research is necessary. Together, these genotype-based tissue expression patterns provide insight for further study of the *MaSWEET* gene family in banana in relevance to plant and fruit development.

### Temporal expression profiles of banana *MaSWEETs* during fruit development and ripening

**Figure 5:**
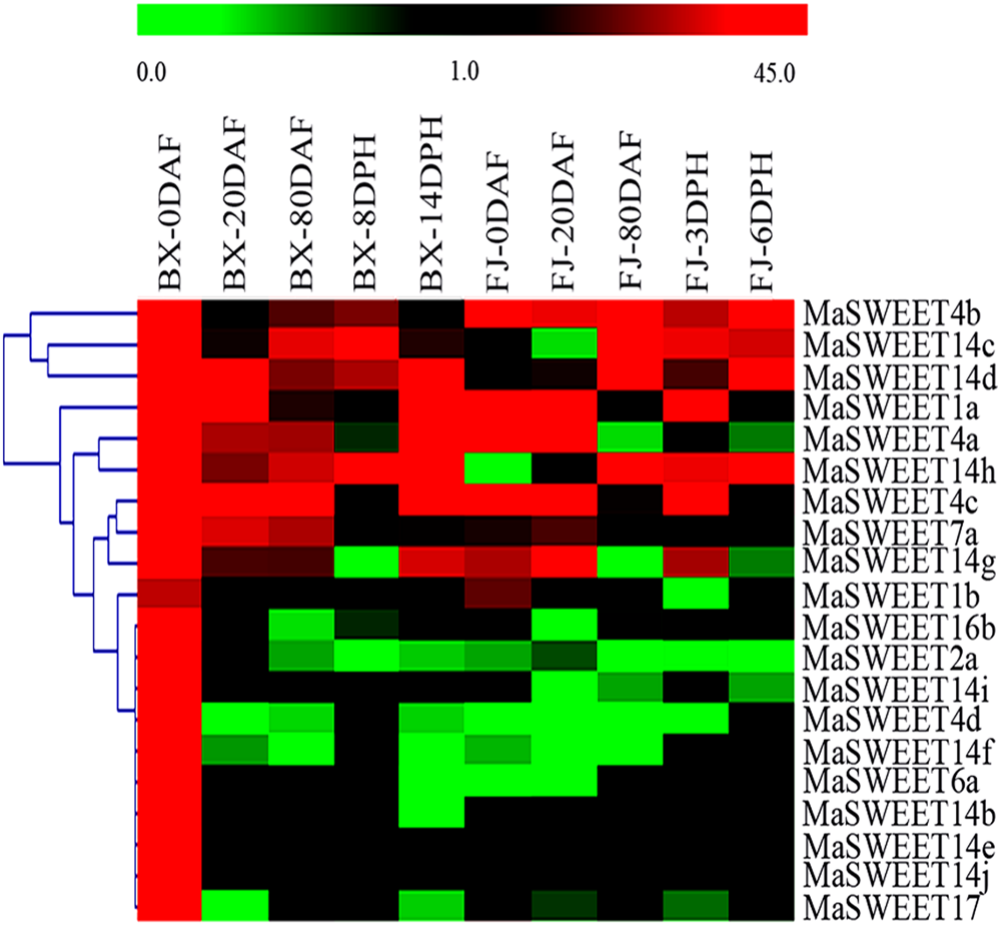
Expression of *MaSWEETs* during different stages of fruit development and ripening in BX and FJ banana varieties. The heat map with clustering was created based on the FPKM value of the *MaSWEETs*. Differences in gene expression changes are shown in color as the scale.

*SWEETs* play an important role in plant development by regulating sugar efflux transport from source- to sink cells^15, 18, 20, 23, 24, 26^. The expression of *MaSWEETs* was detected in fruits sampled 0, 20, and 80 d after emergence from the pseudostem (DAF) in both BX and FJ genotypes, 8 and 14 d postharvest (DPH) from BX, and 3 and 6 DPH from FJ (Fig. 5 and Supplementary Table S5). Twenty of the 25 *MaSWEETs* were expressed during fruit development and ripening of BX and FJ bananas, taken together.

In BX, 20 (80%), 14 (56%), 14 (56%) *MaSWEETs* were expressed 0, 20, and 80 DAF, respectively; 11(44%) and 16 (64%) were expressed 8 and 14 DPH, respectively. Among those expressed, 20 (100%), 8 (57%), 9 (64%), 3 (27%), and 7 (44%) genes were highly expressed (FPKM > 10) at 0, 20, and 80 DAF and 8 and 14 DPH, respectively. Additionally, 3 genes (*MaSWEET*-*14c*, -*14d*, and -*14h*) exhibited high level of expression (FPKM > 10) throughout the entire fruit development and ripening.

In FJ, 16 (64%), 16 (64%), and 13 (52%) *MaSWEETs* were expressed at 0, 20, and 80 DAF, respectively; while 14 (56%), and 11 (44%) were expressed at 3 and 6 DPH, respectively. Among them, 7 (44%), 7 (44%), 5 (39%), 7 (50%), and 4 (36%) genes showed high level of transcript accumulation (FPKM > 10) at all the 5 tested stages, respectively. Furthermore, *MaSWEET4b* exhibited constantly high expression (FPKM > 10) throughout the entire fruit development and ripening.

A comparison of *MaSWEET* expression profiles at different stages of fruit development and ripening in BX and FJ indicated that out of 25 genes, 11 (55%) were expressed at all stages tested in BX, and 10 (50%) were expressed at all stages tested in FJ. Generally, 6 *MaSWEET* (*MaSWEET*-*1a*, -*4a*, -*4b*, -*4c*, -*7a*, and -*14g*) expression profiles were similar between BX and FJ genotypes, with a greater number of highly expressed genes (FPKM > 10) at early stage of fruit development compared to later stages, suggesting *MaSWEETs* may play an important role in fruit formation and initial development regardless of the genotype in banana, despite of higher number of genes being expressed in BX than FJ. Twenty *MaSWEETs* in BX were highly expressed (FPKM > 10), while only 7 (*MaSWEET*-*1a*,-*1b*, -*4a*, -*4b*,-*4c*, -*7a*, and -*14g*) showed similarly high expression (FPKM > 10) in FJ, at the beginning of pseudostem emergence (0 DAF). Moreover, at post-harvest ripening stage, 5 genes (*MaSWEET*-*1a*, -*4a*, -*4c*, -*14g*, and -*14h*) showed higher expression (FPKM > 10) at 14 DPH in BX than in FJ. These findings imply *MaSWEETs* have significant transcriptional response during early fruit development and post-harvest ripening in BX bananas.

### Expression profiles of banana *MaSWEETs* under cold, salt, and osmotic stresses

**Figure 6:**
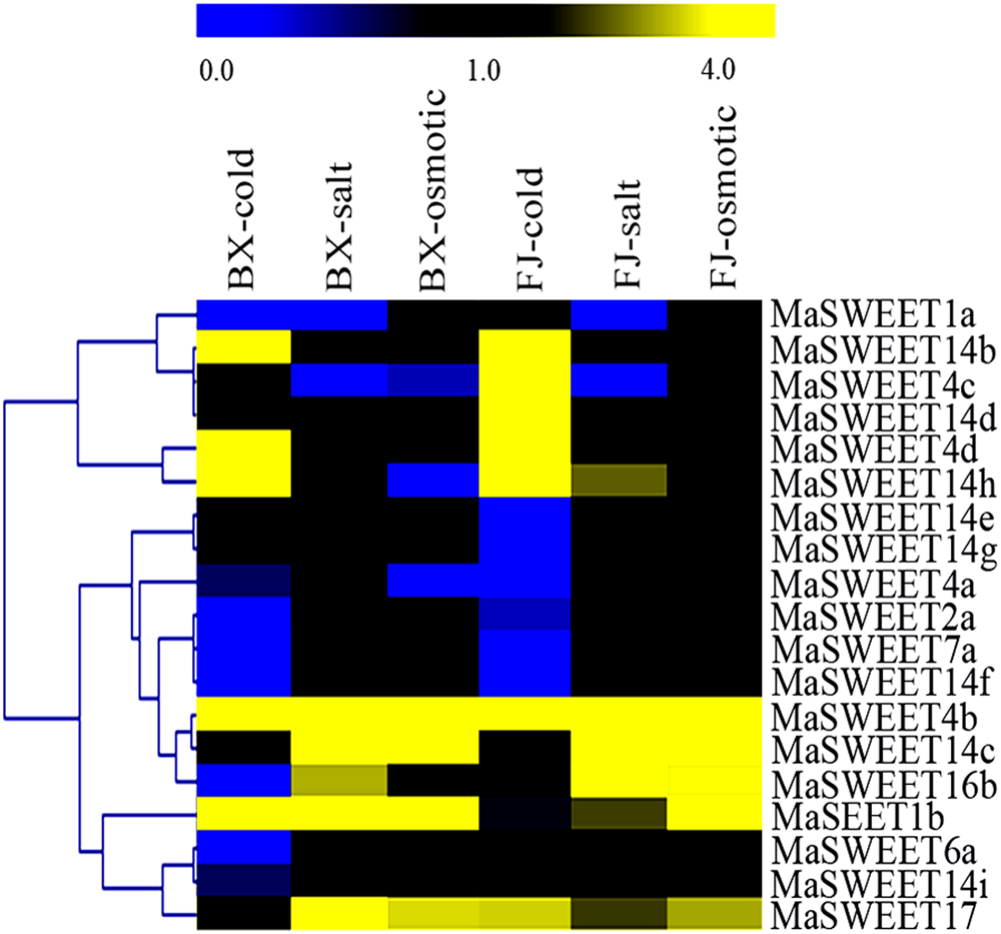
Expression of *MaSWEETs* in response to cold, salt, and osmotic stresses in BX and FJ banana varieties. The heat map with clustering was created based on the FPKM value of the *MaSWEETs*. Differences in gene expression changes are shown in color as the scale.

*SWEET* sugar transporters play important roles in plant responses to abiotic stresses. Thus, *MaSWEET* expression patterns in BX and FJ banana leaf in response to cold, salt, and osmotic treatments were analyzed by RNA-sequencing (RNA-seq) assays (Fig. 6 and Supplementary Table S6). Overall, a total of 17 of the all 25 (90%) *MaSWEETs* showed variable levels of transcription after abiotic stress treatments in BX and FJ leaves.

In BX, the expression of 17 (90%), 7 (37%), and 8 (42%) *MaSWEETs* was induced by cold, salt, and osmotic stresses, respectively. Among them, 5 (29%), 5 (71%), and 4 (50%) were up-regulated (FPKM > 2.0), while 7 (41%), 2 (29%), and 3 (38%) were down-regulated (FPKM < 0.5) by cold, salt, and osmotic stresses, respectively. Additionally, *MaSWEET*-*1b* and -*4b* were significantly upregulated (FPKM > 10) and 3 (*MaSWEET*-*6a*, -*7a*, and -*14f*) were significantly downregulated (FPKM < 0.05) by all three stresses in BX.

In FJ, the expression of 17 (90%), 8 (42%), and 6 (32%) *MaSWEETs* was induced by cold, salt, and osmotic stresses, respectively. Among them, 7 (41%), 6 (75%), and 5 (83%) were upregulated (FPKM > 2); 6 (35%), 2 (25%), and none were downregulated (FPKM < 0.5) by cold, salt, and osmotic stresses, respectively. In addition, 4 (*MaSWEET*-4b, -*4c*,-*4d* and -*14b*), 2 (*MaSWEET*-*4b* and -*14c*), and 2 (*MaSWEET*-*4b* and -*14c*) genes showed significant upregulation (FPKM > 10) by cold, salt, and osmotic stresses, respectively. Furthermore, *MaSWEET4b* was significantly upregulated (FPKM > 10), while *MaSWEET14g* was significantly downregulated (FPKM < 0.05) by all the three stress treatments in FJ.

These results clearly demonstrated that more *MaSWEET* genes were significantly up-regulated in leaf (FPKM > 10) in FJ than BX by cold stress. In addition, the number of genes that were induced to up-regulated express in response to salt and osmotic stresses was also higher in FJ than in BX.

### Expression profiles of banana *MaSWEETs* in response to Foc 4 TR4 infection

**Figure 7:**
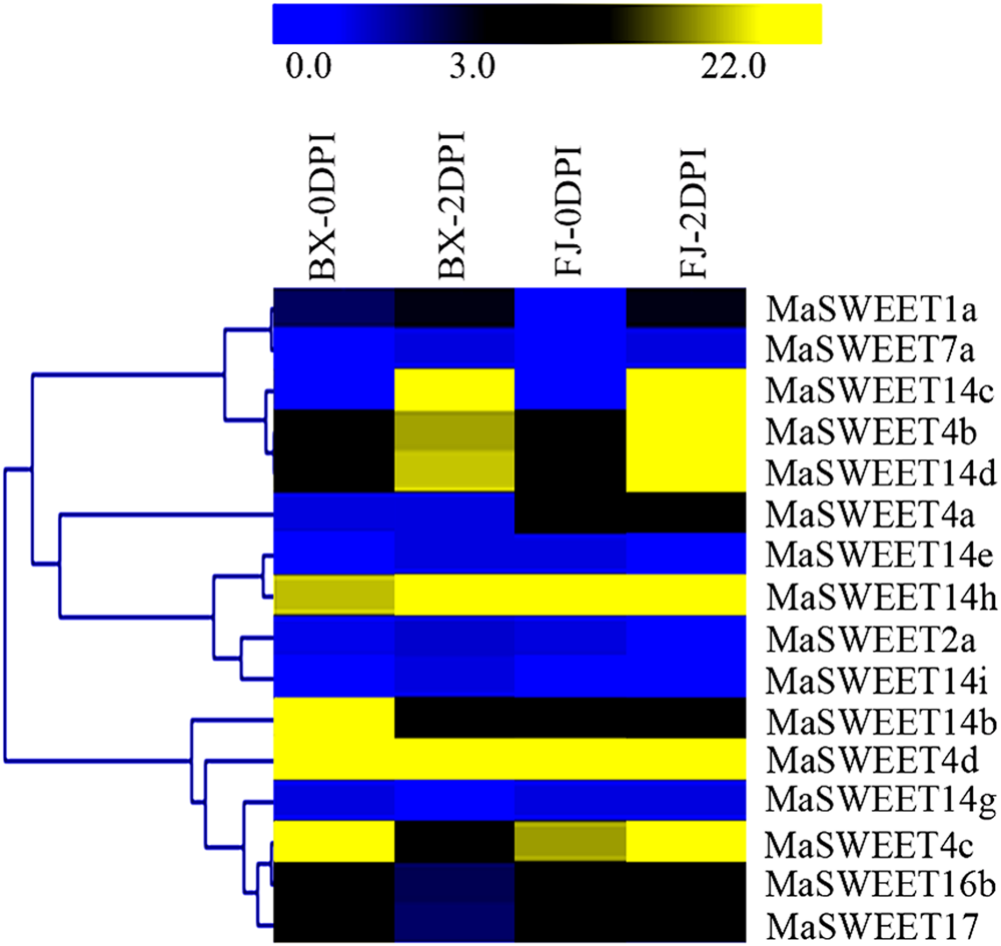
Expression of *MaSWEETs* in response to fungal disease in BX and FJ banana varieties. The heat map with clustering was created based on the FPKM value of the *MaSWEETs*. Differences in gene expression changes are shown in color as the scale.

To obtain insight into the potential functional roles of *MaSWEET* genes in defense against fungal diseases in banana, *MaSWEET* gene expression was analyzed in the entire root system of BX and FJ plants harvested at 0 and 2 d post-infection (DPI). As shown in Fig. 7 and Supplementary Table S7, 16 out of all the 25 *MaSWEET* genes showed transcriptional changes after Foc 4 TR4 infection in both BX and FJ genotypes.

In BX, 9 (56%) and 13 (81%) *MaSWEETs* were expressed 0 and 2 DPI, respectively, with 4 (44%) and 5 (39%) at each respective DPI showing high expression levels (FPKM > 10). A total of 5 genes (*MaSWEET*-*4b*, -*4d*, -*14c*, -*14d*, and -*14h*) exhibited obvious up-regulation at 2 DPI since infection (5-, 1.6-, 32-, 3- and 6-fold increases, respectively). 4 genes (*MaSWEET*-*4c*, -*14b*, -*16b*, and -*17*) were down-regulation at 2 DPI after infection (14-, 15-, 9- and 3-fold decreases, respectively).

In FJ, 11 (69%) and 10 (63%) *MaSWEETs* were expressed at 0 and 2 DPI, respectively, with 5 (46%) and 6 (60%) showing high expression (FPKM > 10) at each respective stage. Additionally, 6 genes (*MaSWEET*-*1a*, -*4b*, -*4d*, -*14c*, -*14d*, and -*14h*) were significantly up-regulated at 2 DPI since infection (6-, 14-, 1.1-, 73-, 12-, and 1.8-fold increase, respectively). 5 genes (*MaSWEET*-*4a*, -*7a*, -*14b*, -*16b*, and -*17*) were down-regulation at 2 DPI after infection (6-, 1-, 11-, 8- and 2-fold decreases, respectively).

The comparison of *MaSWEET* expression profiles at distinct stages of Foc 4 TR4 treatment (0 and 2 DPI) indicated that 16 genes were expressed at both stages tested in BX and FJ. Generally, the *MaSWEET* expression profiles were similar between BX and FJ root systems, with a greater number of highly expressed *MaSWEET* genes at 2 DPI than 0 DPI, indicating *MaSWEETs* play important roles in response to Foc 4 TR4 infection. Further, great number of *MaSWEET* genes showed higher level of expression in FJ than BX. A total of 4 genes (*MaSWEET*-*4b*, -*4c*, -*14c*, and -*14d*) showed higher expression (FPKM > 10) at 2 DPI in FJ compared with BX. In addition, *MaSWEET*-*4d* and -*14h* were highly expressed (FPKM > 10) in all tested stages of BX and FJ, suggesting that these two genes may play extensive and vital roles in the response to Foc 4 TR4 stresses in banana.

### Validation of the differentially expressed banana *MaSWEET* by qRT-PCR

Based on RNA-seq data, *MaSWEET*-*4b* and -*14c* were highly expressed (FPKM > 4) in all tested banana tissues including root, leaf, and fruit, while differential expression between the two genotypes was detected in *MaSWEET*-*1a* and -*14g*. *MaSWEET*-*4b* and -*14d* were highly expressed (FRKM > 2) in all tested fruit development and ripening stages in both BX and FJ genotypes, while *MaSWEET*-*14h* and -*14g* showed genotypic variation of expression at these stages. *MaSWEET*-*4b* and -*14c* were upregulated by cold, salt, and osmotic stresses in BX and FJ, while *MaSWEET*-*4c* and -*14d* showed genotypic variations in response to each abiotic stress. Furthermore, *MaSWEET*-*4d* and -*14h* were upregulated by Foc 4 TR4 infection in BX and FJ, whereas *MaSWEET*-*4c* and -*14b* exhibited genotype-specific response to Foc 4 TR4 infection.

**Figure 8:**
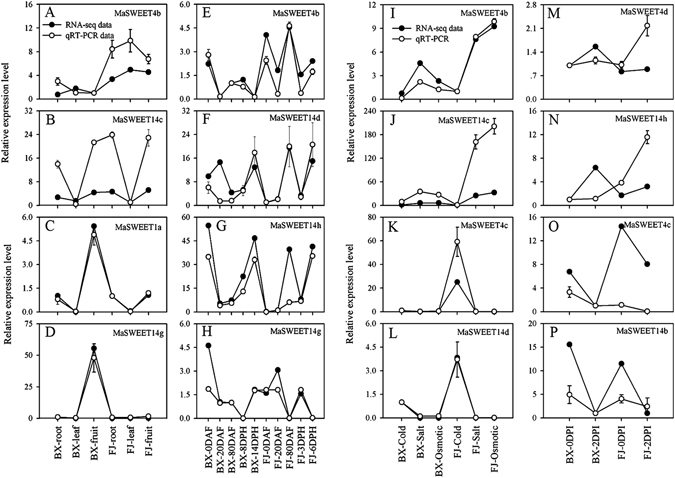
Relative expression of *MaSWEETs* in BX and FJ banana varieties by qRT-PCR. (**A**–**D**) Expression of *MaSWEET*-*4b*, -*14c*, -*1a* and -*14g* in different organs. (**E**–**H**) Expression of *MaSWEET*-*4b*, -*14d*, -*14h* and -*14g* at different stages of fruit development and ripening. (**I**–**L**) Expression of *MaSWEET*-*4b*, -*14c*, -*4c* and -*14d* in response to cold, salt and osmotic stresses. (**M**–**P**) Expression of *MaSWEET*-*4d*, -*14h*, -*4c* and -*14b* in response to fungal infection. Data are presented as means ± standard deviations of *n* = 3 biological replicates.

For verification, the above mentioned differentially expressed *MaSWEETs* were selected for qRT-PCR analysis to validate RNA-seq data. After normalization, all the examined *MaSWEETs* with the exception of *MaSWEET*-*4b* and -*14c* in BX leaves, *MaSWEET4b* in BX 0 DAF, *MaSWEET14d* in BX 14 DPH and FJ 6 DPH, and *MaSWEET*-*4d* and -*14h* in FJ 0 and 2 DPI showed trends consistent with the RNA-seq analysis (Fig. 8). The correlation coefficient between RNA-seq and qRT-PCR data ranged from 0.7493 to 0.9997 (Supplementary Table S8), except *MaSWEET*-*4d*, -*14h*, and -*4c* in 0 and 2DPI of BX and FJ (Supplementary Table S8M,N, and S8O). These results indicate that RNA-seq analysis supplied suitable expression results for both banana varieties.

### SWEET family interaction networks and their co-expression in early fruit development stages and under abiotic/biotic stresses﻿

**Figure 9:**
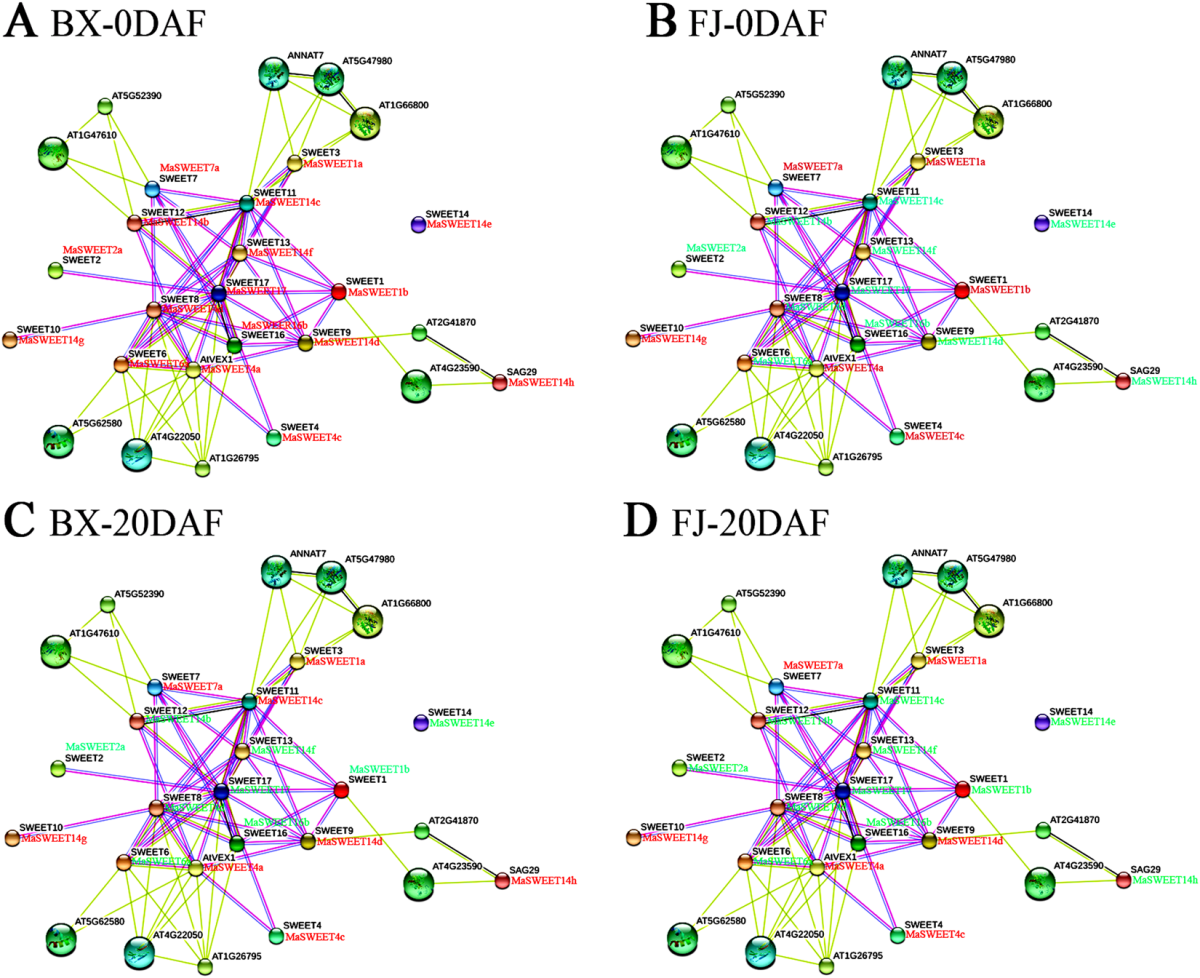
Interaction network and co-expression analyses of *MaSWEETs* at 0 DAF (**A**,**B**) and 20 DAF (**C**,**D**) in BX and FJ banana varieties and related genes in *Arabidopsis*. Line thickness relates to combined score. Genes marked in red show upregulation in BX and FJ. Genes marked in green show downregulation in BX and FJ.

Studying gene family interaction networks is very useful for investigating potential gene functions^40^. *SWEET* genes have been documented as playing active roles in plant development and abiotic/biotic stresses in many species^4, 15, 28–30, 33^. However, due to the paucity of such reports in banana, we investigated the potential MaSWEET protein interaction and co-expression networks to aid future studies investigating their biological function based on experimentally validated interactions. An AtSWEET network was constructed, and 27 interactive proteins (with high confidence; score > 0.9), including 17 SWEETs and 10 other interactive proteins [alcohol dehydrogenase-like protein (AT1G66800), photoassimilate-responsive protein 1 (AT5G52390), WD40 domain-containing protein (AT1G47610), remorin-like protein (AT2G41870), tyrosine aminotransferase family protein (AT4G23590), self-incompatibility protein S1-like protein (AT1G26795), armadillo/beta-catenin-like repeat family protein (AT5G62580), BAHD acyltransferase (AT5G47980), annexin 7 (AT5G10230), and aspartyl protease family protein (AT4G22050), were identified with STRING^41^. Then, orthologs of these interaction proteins in banana were identified with reciprocal BLASTP analyses, and the expression profiles of these genes in BX and FJ at early development stages (0 and 20 DAF) were extracted from RNA-seq data sets (Fig. 9 and Supplementary Tables S9 and S10). At 0 DAF in BX, 38 gene pairs showed uniform up-regulation, but down-regulated gene pairs were not detected (Fig. 9A and Supplementary Table S10). At 0 DAF in FJ, only one gene pair (AtVEX1:MaSWEET4a-SWEET4:MaSWEET4c) showed uniform up-regulation, while 19 pairs showed uniform down-regulation (Fig. 9B and Supplementary Table S10). At 20 DAF, 5 gene pairs were up-regulated in BX, whereas 2 were up-regulated in FJ (Fig. 9C,D and Supplementary Table S10). Additionally, 18 gene pairs showed negative correlation in FJ at 0 DAF (Fig. 9B and Supplementary Table S10). Collectively, interaction network and co-expression analyses indicate the crucial roles of MaSWEET member-mediated networks in early fruit development, with more gene pairs found uniformly up-regulated in BX than in FJ at 0 DAF.

**Figure 10:**
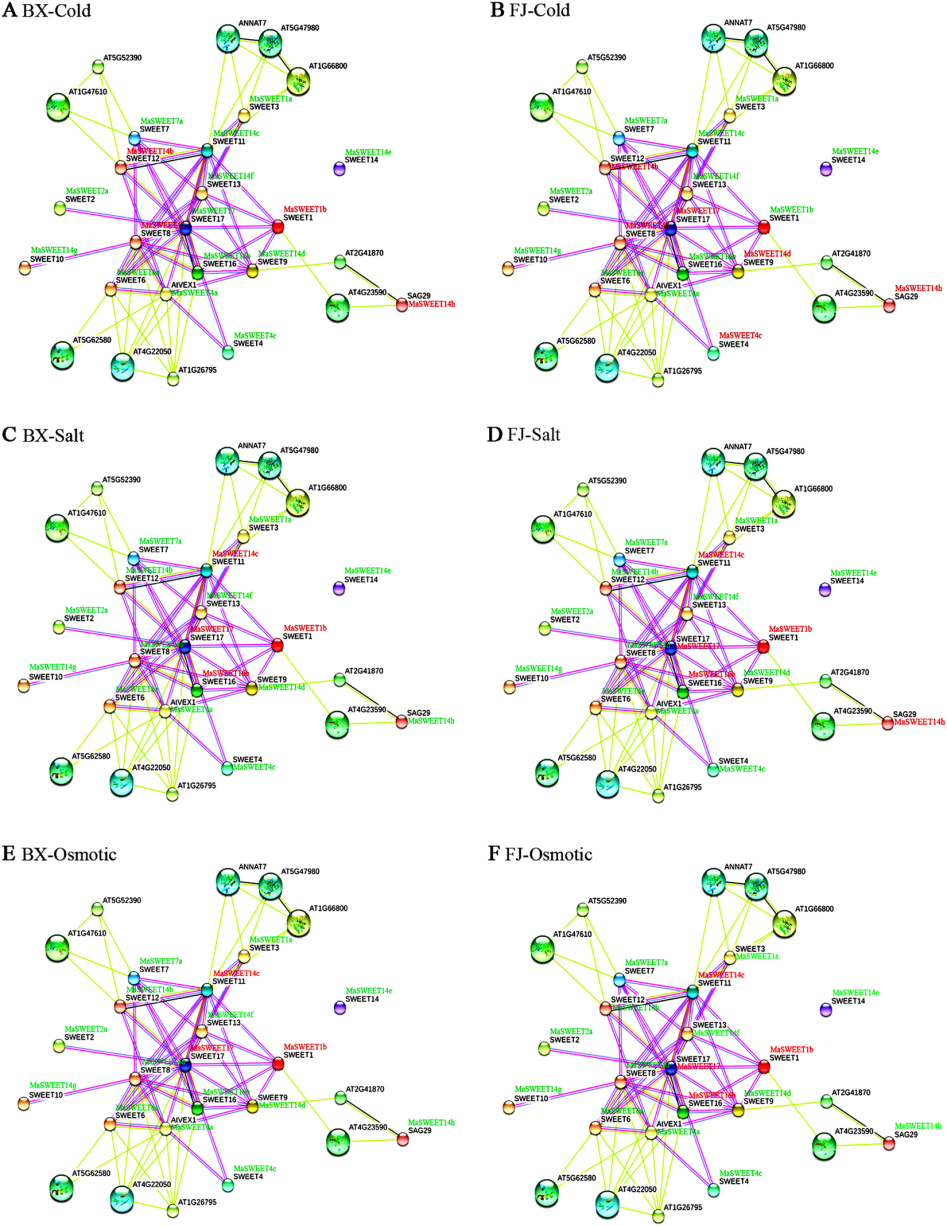
Interaction network and co-expression analyses of *MaSWEETs* under cold (**A**,**B**), salt (**C**,**D**), and osmotic (**E**,**F**) stresses in BX and FJ banana varieties and related genes in *Arabidopsis*. Line thickness relates to combined score. Genes marked in red show upregulation in BX and FJ. Genes marked in green show downregulation in BX and FJ.

Under cold stress, 23 gene pairs showed uniform down-regulation in BX, but up-regulated gene pairs were not detected (Fig. 10A and Supplementary Table S11); in FJ, 3 gene pairs showed uniform up-regulation, while 12 pairs showed uniform down-regulation (Fig. 10B and Supplementary Table S11). Under salt stress, 5 gene pairs were up-regulated in BX, whereas 14 pairs were down-regulated (Fig. 10C and Supplementary Table S11); in FJ, the number of up- and down-regulated gene pairs is consistent with that in BX (Fig. 10D and Supplementary Table S11). Under osmotic stress, 3 gene pairs showed uniform up-regulation in BX, 18 gene pairs were down-regulation (Fig. 10E and Supplementary Table S11); in FJ, 5 gene pairs showed uniform up-regulation, while 14 pairs showed uniform down-regulation (Fig. 10F and Supplementary Table S11). Collectively, interaction network and co-expression analyses show the important roles of MaSWEET member-mediated networks under abiotic stresses, with more gene pairs found uniformly up-regulated in FJ than in BX under cold and osmotic stresses.

**Figure 11:**
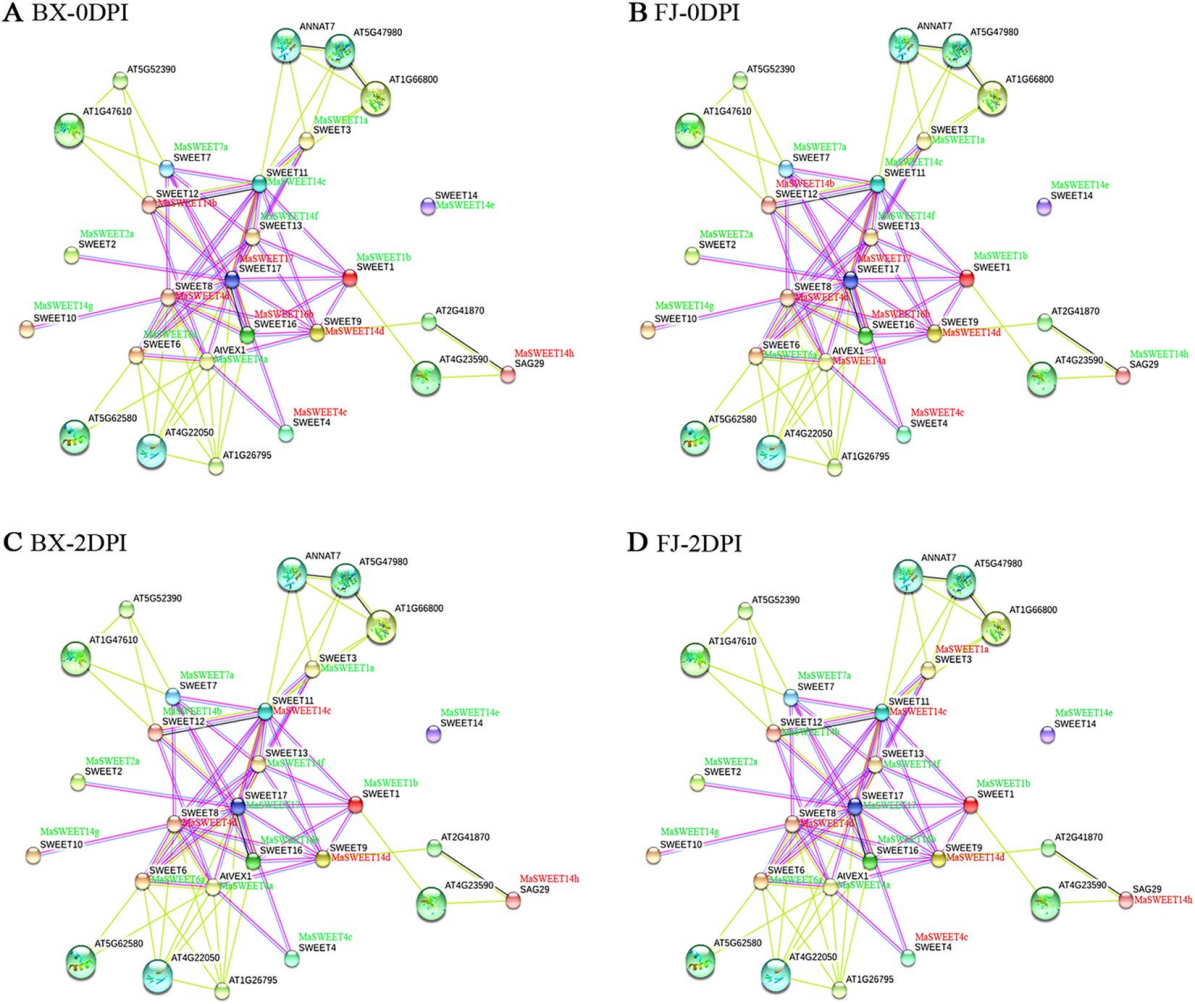
Interaction network and co-expression analyses of *MaSWEETs* at 0 DPI (**A**,**B**) and 2 DPI (**C**,**D**) in BX and FJ banana varieties and related genes in *Arabidopsis*. Line thickness relates to combined score. Genes marked in red show upregulation in BX and FJ. Genes marked in green show downregulation in BX and FJ.

At 0 DPI, 7 gene pairs showed uniform up-regulation in BX, 11 gene pairs were down-regulation (Fig. 11A and Supplementary Table S12); in FJ, 12 gene pairs showed uniform up-regulation, while 9 pairs showed uniform down-regulation (Fig. 11B and Supplementary Table S12). At 2 DPI since infection, 3 gene pairs showed uniform up-regulation in BX, 18 gene pairs were down-regulation (Fig. 11C and Supplementary Table S12); in FJ, 4 gene pairs showed uniform up-regulation, while 15 pairs showed uniform down-regulation (Fig. 11D and Supplementary Table S12). Taken together, these results show the potential functional roles of *MaSWEET* genes in defense against fungal diseases in banana, with more gene pairs found uniformly down-regulated in BX and FJ at 2 DPI since infection.

## Discussion

In spite of the economic and social importance of bananas, research on banana plants has generally be slow relative to other crops, especially with respect to fruit development and responsiveness to abiotic and biotic stresses^37^. The SWEET family represents a novel type of sugar transporter that has participated in the regulation of plant growth, development, and stress responses in many plant species^4, 15, 18, 20, 22^. Using the genome wide identification approach, we have identified 25 *MaSWEET* genes in *M*. *acuminata*, and made a comparative analysis in two genotypes including BX and FJ. These genes were classified into four subfamilies (Clades I-IV) according to their phylogenetic evolutionary relationship (Fig. 1), which was consistent with the classification of *SWEETs* in *A*. *thaliana* and *O*. *sativa* ^14, 22^. However, there are also discrepancies in the size of subfamilies, for instance in Clade III, a staggering 10 members of *MaSWEET14* was found, which is in sharp contrast to only one member in *AtSWEET* and *OsSWEET* (Fig. 1). In addition, the number of duplicated gene pairs (Ka/Ks ratio <1 or >1) was different between AAA and AAB, AAA and Arabidopsis, and AAA and rice (Supplementary Table S2). This may suggest a broad number event of gene duplication and divergence occurred in *MaSWEET14* members during banana evolutionary history compared to *A*. *thaliana* and *O*. *sativa*.

These evolutionary and phylogenetic analyses were supported by gene structure and conserved motif analyses. Gene structure analysis indicated that *MaSWEETs* within each Clade shared a similar exon-intron organization (Fig. 2). For example, each member of Clades I, III (except *MaSWEET*-*14h* and -*14b*) and IV harbors 5 introns, whereas Clade II members contain fewer (4 introns). According to a previous report in *O*. *sativa*, the rate of intron loss is faster than that of intron gain after segmental duplication^42^. We therefore propose that Clades I, III, and IV might contain the original ancestor *MaSWEET* genes, and Clade II might be derived by gene duplication and divergence with subsequent intron loss. This structural feature of *MaSWEETs* has also been observed in other plant species, such as *S*. *lycopersicum* ^18^ and *G*. *max* ^22^. Moreover, conserved motif analysis indicated that all the *MaSWEETs* harbored the seven conserved TMDs, while gene members within each subfamily shared other more specific motifs (Fig. 3). Such an observation is consistent with other plants, such as *A*. *thaliana* ^15^, *S*. *lycopersicum* ^18^, and *O*. *sativa* ^20^.

The fruit development and ripening processes are crucial for banana yield and quality. The *SWEET* family has been reported to participate in the fruit development process in many plant species, such as *M*. *domestica* ^24^, *S*. *lycopersicum* ^18^, and *Vitis vinifera* ^4^. In tomato, expression levels of *SWEET12C* and *SWEET14* were relatively high in ripening fruit^43^. SWEETs putatively control sucrose accumulation in sweet sorghum stems^19^. However, whether *MaSWEETs* in banana have also participated in fruit development and postharvest ripening, so far remained elusive. In the current study, we found that more than 80% *MaSWEETs* were expressed during the five developmental and ripening stages tested in BX and FJ varieties, and more than 27% of them were highly expressed (FPKM > 10) at each stage (Fig. 5), implying that *MaSWEETs* are extensively involved in banana fruit development and ripening. Interestingly, *MaSWEET* expression patterns at a certain stage was substantially different between genotypes, for instance, 20 *MaSWEETs* was highly expressed (FPKM > 10) in BX compared to only 7 in FJ, in the initial stage of pseudostem emergence (0 DAF). The greater number of highly expressed genes during early development in BX versus FJ may be explained by the genome variations between these two genotypes. The majority of edible cultivated bananas are triploid, originating from intra- or interspecific hybridization between the diploid *M*. *acuminata* (A genome) and *M*. *balbisiana* (B genome), producing AAA, AAB, and ABB genotypes among others^44, 45^. The BX genotypes, with AAA genome, is known to produce high yield and quality fruit with long fingers and extended shelf life and storage period compared to the FJ genotype with AAB genome^37, 46^. It is tempting to assume that the increase of *MaSWEET* expression during early stage of fruit development augments sugar transport ability, thereby enhancing quality and yield of banana fruit. Furthermore, interaction network and co-expression analyses showed more gene pairs were uniformly up-regulated in BX than in FJ in the early stages of fruit development (Fig. 9). Such findings in this study is consistent with previous studies suggesting that the A-genome harbors more genes that are important for banana yield and quality and could be used as a target in breeding programs^37, 46^.

Bananas are large, monocotyledonous, herbaceous plants that are extremely sensitive to abiotic stress, such as those imposed by drought, salt, or cold conditions^46^. However, the response of banana to abiotic stresses is largely inadequate^47^. The expression of *SWEETs* in various plant species reportedly changes in response to multiple abiotic stresses^18, 32, 48^. For example, in *S*. *lycopersicum*, Class II *SlSWEETs* were found to be upregulated several fold in leaves by sugar, salt, and temperature stress treatments^18^. Under cold stress conditions, *AtSWEET16* overexpression give rise to plants with increased freezing tolerance compared to wild type, probably due to a greater accumulation of vacuolar sucrose and glucose^32^. *AtSWEET15*, also known as *SAG29*, was strongly induced during senescence and abiotic stresses, such as cold, high salt, and drought treatments^48^. Under high salt conditions, *AtSWEET15*-overexpressing lines exhibited reduced root growth and cell viability compared to control plants^48^. Under these stress conditions, the expression of *SWEETs* tended to promote sugar accumulation in plant vacuoles and change plant organ developmental states. In the present study, 76% *MaSWEETs* showed transcriptional changes after abiotic stress treatment, including osmotic, salt, and cold stresses, in both BX and FJ genotypes (Fig. 6). Previous gene expression analysis have shown increased *SWEET* transcription when subjected to cold, drought, and salt abiotic stresses in other plants, such as *S*. *lycopersicum* ^18^, *A*. *thaliana* ^32^ and *O*. *sativa* ^26^. These results indicate that *SWEET* genes exhibit an extensive response to abiotic stresses. Comparative analysis clearly demonstrated that more *MaSWEET* genes were significantly upregulated in FJ than in BX tissues subjected to cold treatments. In salt and osmotic stresses, the number of genes that were induced to up-regulated express was also higher in FJ than in BX (Fig. 6). B-genomecontaining banana varieties have previously been reported to confer strong tolerance to abiotic stress^45, 46^. In addition, numerous studies have confirmed that *SWEETs* are involved in maintaining sugar homeostasis in plant organs, facitating adaptation to low temperatures^27, 48^. Taken together, it is reasonable to propose that significant upregulation of *MaSWEETs* in FJ banana plants subjected to cold stress could be attributable to thermal tolerance.

Currently, banana production has been devastated by fungal infestations caused by Foc TR4^49^. However, it remains unresolved whether *SWEET* genes play a role in banana disease resistance. In cassava, the bacterial blight *Xanthomonas axonopodis* was found to directly induce *MeSWEET10a* transcription^50^. Similarly, expression of *OsSWEET14* confers resistance against bacterial blight in wild *O*. *sativa* ^51^. Recently, it was reported that in addition to *Xanthomonas*, other bacterial and fungal pathogens, such as *Botrytis cinerea* and some mycorrhizal fungi, also modulate expression of plant *SWEETs* ^4, 52^. In the current study, we found transcriptional up-regulation of 3 *MaSWEET* genes (*MaSWEET*-*4c*, -*4d*, and -*14h*) in response to Foc TR4 infection in both BX and FJ bananas, suggesting a possible crucial role for these *MaSWEETs* to play in response to fungal infections in banana. Additionally, higher number of *MaSWEET* genes were highly expressed in FJ than in BX, suggesting that the B-genome which is not present in BX, may also be involve in protection against the biotic stress in banana.

In conclusion, we identified 25 *MaSWEET* genes from the *M*. *acuminata* genome, which could be classified into four clades following phylogenetic analysis, gene structure, and conserved protein motif analyses. Spatial and temporal expression profiles of *MaSWEETs* in two triploid banana varieties revealed that *MaSWEETs* may have distinct functions during the fruit development and ripening. The expression patterns of *MaSWEETs* in response to both abiotic and biotic stresses indicate that they are also involved in stress signaling pathway regulation, especially in B-genomecontaining banana varieties. Furthermore, interaction networks and co-expression assays indicated the strong transcriptional response of banana *SWEET*-mediated networks in early fruit development and under abiotic/biotic stresses of A-genomecontaining banana varieties, suggesting *MaSWEETs* enhance sugar transport during early fruit development and under abiotic/biotic stresses. These results serve to advance the current understanding of *SWEET* functional characteristics in banana developmental processes and stress responses and lay a solid foundation for further genetic improvement of banana quality, yield, and resistance to both biotic and abiotic stresses.

## Methods

### Plant materials and treatments

BX [*M*. *acuminate* L. AAA group, cv. Cavendish] is a triploid variety of banana widely cultivated in tropical and subtropical regions in China, which produces a high yield of high quality fruits fit for long-term storage. FJ [*M*. *acuminata* AAB group] is also a triploid variety cultivated in the Guangdong and Hainan provinces of China, which had good fruit flavor, ripens quickly, and exhibits high tolerance to abiotic stresses. Samples of both genotypes were obtained from the banana plantation at the Chinese Academy of Tropical Agricultural Sciences (Danzhou, Hainan, China). Five-leaf stage young BX and FJ banana seedlings with uniform growth were grown in soil at 28 °C with 70% relative humidity and 16 h light/8 h dark cycle with 200 μmol·m^−2^·s^−1^ light intensity.

For salt and osmotic stress treatments, 5-leaf stage banana seedlings were irrigated with 300 mM NaCl and 200 mM mannitol for 7 d, respectively. For cold treatment, banana seedlings were maintained at 4 °C for 22 h. For examination of banana fruit development, fruits were collected 0 (budding), 20 (cutting flower), and 80 (ready for harvest) DAF from both BX and FJ plants. To assess banana fruit postharvest ripening, BX fruits stored for 8 and 14 DPH and FJ fruits stored for 3 and 6 DPH, which represent fruits ripening stages of “more green than yellow” and “full yellow” according to Pua *et al*.^53^ in each variety, were sampled. For Foc TR4 treatments, roots of 5-leaf stage banana seedlings were dipped in a Foc TR4 spore suspension of 1.5 × 10^6^ condia/mL. The entire root system was harvested at 0 and 2 DPI, immediately frozen in liquid nitrogen, and stored at −80 °C until expression analysis.

### Identification and phylogenetic analyses of *MaSWEET* genes in banana

Whole *M*. *acuminata* banana protein sequences were downloaded from the banana genome database (http://banana-genome.cirad.fr/)^35^. SWEET amino acid sequences from *A*. thaliana and *O*. *sativa* were obtained from the TAIR (http://www.arabidopsis.org/) and RGAP (http://rice.plantbiology.msu.edu/) databases, respectively. HMM profiles of the SWEET domain (PF00170) (http://pfam.sanger.ac.uk/) were used to query the predicted SWEET proteins in the banana genome databases using HMMER software (HMMER: http://hmmer.wust.edu/). BLAST analyses were also used to identify the predicted banana SWEETs using all *A*. *thaliana* and *O*. *sativa* SWEETs as queries. A conserved domain search of the potential banana SWEETs was further validated using CDD (http://www.ncbi.nlm.nih.gov/cdd/) and PFAM (http://pfam.sanger.ac.uk/) databases. The accession number of all identified banana SWEETs are listed in Table S1. Phylogenetic analysis of SWEET amino acids sequences from banana, *A*. *thaliana* and *O*. *sativa* were carried out using Clustal X2.0 and MEGA5.0 software with bootstrap values for 1000 replicates. The Ka/Ks ratio was used to show the selection pressure for the duplicate genes^54^.

### Protein properties and gene structure analysis

Molecular mass and isoelectric points of MaSWEET proteins were predicted using the ExPASy database (http://expasy.org/). Motifs of MaSWEET proteins were analyzed using MEME software (http://meme.nbcr.net/meme/cgi-bin/meme.cgi) and annotated by InterProScan database search (http://www.ebi.ac.uk/Tools/pfa/iprscan/). Structural features of *MaSWEET* genes were identified using Gene Structure Display Server software (http://gsds.cbi.pku.edu.cn/). The interactions between SWEETs and their transcriptional factors were determined using STRING (http://string-db.org/) with a confidence score >0.9 and Cytoscape software.

### Transcriptomic analysis

To investigate the transcriptional accumulation of *MaSWEET* genes in different organs of the BX and FJ varieties, roots, leaves, and fruits at 80 DAF were collected. Banana fruits at different development stages, i.e. 0, 20, and 80 DAF, were collected to assess the expression patterns of banana *MaSWEETs* during fruit development. To examine the expression of *MaSWEETs* during postharvest ripening processes, fruits at 8 (more green than yellow) and 14 (full yellow) DPH in BX and at 3 (more green than yellow) and 6 (full yellow) DPH in FJ were collected. The 5-leaf stage banana seedlings were treated with 200 mM mannitol for 7 days, 300 mM NaCl for 7 days, low temperature (4 °C) for 22 h, or Foc TR4 spore suspension (1.5 × 10^6^ condia/mL) dipping roots for 2 days, respectively. All samples were collected and frozen quickly in liquid nitrogen and stored at −80 °C for RNA extraction and transcriptome analysis. Each sample contains three biological replicates.

Total RNA from each sample was extracted using a plant RNA extraction kit (Tiangen Biotech, Beijing, China), and then cDNA libraries constructed according to the protocols supplied by Illumina. RNA-seq was performed with an Illumina GAII following the manufacturer’s instructions, with two replicates per sample. The sequencing depth was 5.34X on average. Adapter sequences in the raw sequence reads and low quality sequences were removed using FASTX-toolkit and FastQC, respectively. The obtained clean reads were mapped to the DH-Pahang genome (*M*. *acuminata*, diploid A genome)^35^. Transcriptome assemblies were performed using Cufflinks^55^. Gene expression levels were calculated as FPKM. Differentially expressed genes were then sequenced from RNA-seq data.

### qRT-PCR analysis

The expression levels of *MaSWEETs* in different tissues, different stages of fruit development and ripening, and in response to cold, salt, osmotic, and Foc TR4 stresses were further assessed by qRT-PCR using a Stratagene Mx3000P detection system (Stratagene, San Diego, CA) with SYBR^®^ Premix *Ex* Taq^TM^ kit (TaKaRa Bio Inc, Otsu Shiga, Japan). Primers that had high specificity and efficiency according to melting curve analysis and agarose gel electrophoresis were used to conduct quantification analysis (Supplementary Table S13). Amplification efficiencies of primer pairs ranged from 0.9 to 1.1, ACTIN (GenBank accession no.EF672732) and UBQ (GenBank accession no. XP009390884.1) were verified as being constitutively expressed and suitable as internal controls were used as reference genes to normalize transcriptional levels of *MaSWEET* genes. Relative expression levels of *MaSWEETs* were analyzed in triplicate and calculated using the 2^−ΔΔ*C*T^ method^56^.

## Electronic supplementary material

Table S1-S13
